# Exploring health systems research and its influence on policy processes in low income countries

**DOI:** 10.1186/1471-2458-7-309

**Published:** 2007-10-31

**Authors:** Adnan A Hyder, Gerald Bloom, Melissa Leach, Shamsuzzoha B Syed, David H Peters, Future Health Systems: Innovations for Equity

**Affiliations:** 1 Johns Hopkins University Bloomberg School of Public Health, 615 North Wolfe Street, Suite E-8132, Baltimore, MD 21205, USA; 2 Institute of Development Studies (IDS), University of Sussex, UK

## Abstract

**Background:**

The interface between research and policymaking in low-income countries is highly complex. The ability of health systems research to influence policy processes in such settings face numerous challenges. Successful analysis of the research-policy interface in these settings requires understanding of contextual factors as well as key influences on the interface. *Future Health Systems (FHS): Innovations for Equity *is a consortium conducting research in six countries in Asia and Africa. One of the three cross-country research themes of the consortium is analysis of the relationship between research (evidence) and policy making, especially their impact on the poor; insights gained in the initial conceptual phase of FHS activities can inform the global knowledge pool on this subject.

**Discussion:**

This paper provides a review of the research-policy interface in low-income countries and proposes a conceptual framework, followed by directions for empirical approaches. First, four developmental perspectives are considered: social institutional factors; virtual versus grassroots realities; science-society relationships; and construction of social arrangements. Building on these developmental perspectives three research-policy interface entry points are identified: 1. Recognizing policy as complex processes; 2. Engaging key stakeholders: decision-makers, providers, scientists, and communities; and 3. Enhancing accountability. A conceptual framework with three entry points to the research-policy interface – policy processes; stakeholder interests, values, and power; and accountability – within a context provided by four developmental perspectives is proposed. Potential empirical approaches to the research-policy interface are then reviewed. Finally, the value of such innovative empirical analysis is considered.

**Conclusion:**

The purpose of this paper is to provide the background, conceptual framework, and key research directions for empirical activities focused on the research-policy interface in low income settings. The interface can be strengthened through such analysis leading to potential improvements in population health in low-income settings. Health system development cognizant of the myriad factors at the research-policy interface can form the basis for innovative future health systems.

## Background

The interface between policymaking and research in low-income countries is highly complex. Health systems research in such settings face a number of challenges including under-investment, lack of human capacity, lack of public demand, inadequate utilization, and poor dissemination of results [1-3]. Mismatch between need for health research and investment has been highlighted and attempts made to address the '10/90' gap including: research capacity strengthening; promotion of research investment; and the establishment of global and national health forums [4,5]. In addition, empirical work on mapping health resource flows and health research systems has progressed [6,7]. At the same time, the search for strategies to "get research findings into policy and practice" has gained momentum and the global literature has called for further exploration in the area of *research to policy *[8]. In particular, engaging decision makers in specific areas of health research, and promoting the use of surveys of decision makers has been advocated [9].

The developed world is the focus of much of the exploration in the arena of research to policy [10,11] and specifically on pathways from research to development of clinical guidelines, or on the use of systematic reviews [12]. A summary of the key challenges to bridging the research-policy gap and strategies to overcome them in the developed world exist [13]. In recent years, some country specific analyses of the research-policy interface in the developing world have also been articulated. These include analyses from: India [14]; Lao PDR [15]; Cambodia [16]; Viet Nam [17]; Thailand [18]; Bangladesh [19]; Nepal [20]; Sri Lanka [21]; Uganda [22]; Kenya [23]; Nigeria [24]; Mali [25]; and Mexico [26]. These analyses either focus on health policy making *in general*, or focus on specific but divergent health issues and the policies that pertain to them.

Despite the relative paucity of empirical work, the World Health Organization [27], the Alliance for Health Policy & Systems Research [28], the Council on Health Research for Development [29], and other organizations have made important contributions to the knowledge base on the research to policy interface [3,6,30]. These contributions are: reliant on the use of small scale, case study approaches; focused on a few health issues; limited to one country or to specific locations within a country; and largely unpublished in the peer-reviewed literature, although important reports and documents have been produced and circulated. The Alliance for Health Policy and Systems Research has produced such a publication, which presents the common conceptual model of a linear process – evidence is generated, findings are made available, and eventually decisions are influenced [31]. In reality the process of evidence translation into decision making within government or other institutions is rather more complex.

Drawing on work on policy processes in other arenas, research and the evidence it generates can be seen differently. Evidence is not the rational, objective, set of facts it is sometimes made out to be; rather it can be seen as *forms of knowledge *– partial perspectives – forwarded by particular people and institutions, and sometimes contested by others [32,33]. Knowledge relevant to health system organization is not just about diseases and technologies (e.g. disease epidemiology, drug or vaccine efficacy), or about nature-society interactions (e.g. social influences on disease pathways). Crucially, the importance of knowledge about society and social institutions, which differs from knowledge about diseases or nature-society interactions, cannot be ignored. More importantly, understanding which knowledge and perspectives come to influence policy, and which are excluded, requires understanding the policy process as non-linear – shaped through politicized negotiations amongst multiple actors [32,34-36]. Furthermore, there is often a process of mutual construction of research and policy, in which policy negotiations shape what kinds of research are funded and carried out, and which are not.

The Research Programme Consortium (RPC), Future Health Systems: Innovations for Equity, funded by the Department for International Development (DFID), United Kingdom hopes to heed the calls made by previous work done in this field, and contribute to both methods and results that are useful for understanding the nexus of research and policy and how that can positively impact the poor [37,38]. The overall goal of the 'research to policy' thematic activities in the consortium is to understand the relationship between research (evidence) and the development of policies, especially their impact on the poor. More specifically this consortium seeks to: document previous *experiences *of decision makers with health research; understand overall *values *placed on health research and evidence by decision makers; define the context and conditions under which decision makers will *demand *health research; identify *characteristics *of health research that make it attractive to decision makers; and explore the existence and performance of *institutional mechanisms *that allow interaction between research evidence and policy development and implementation at national and sub-national level.

Given the above overarching and specific thematic goals, this paper aims to provide the background, conceptual framework, and key research directions for such types of empirical activities in low income settings. Firstly, four key developmental perspectives that are particularly relevant contextual factors to the research-policy interface are considered. Second, three entry points for the research-policy interface in the developing world are discussed. Third, a conceptual framework for use in the analysis of the research-policy interface is proposed. Fourth, potential study designs and methodologies required in responding to the identified knowledge gaps in the research-policy interface in low-income countries are identified. Finally, the overall added value of the entire approach is considered.

## Discussion

### The context of health decisions

Analysis of policy processes involves understanding not only the mechanics of decision-making and implementation, but also more complex underlying practices of policy framing. Framing here refers to how boundaries are drawn around problems, how policy problems are defined, and what is included and excluded. Problems and solutions related to health systems may include disease problems and proposed solutions in health interventions. However, problems and solutions related to health systems also encompass broader questions about health system organization, and new forms of social, political or economic arrangements. Understanding and addressing these broader dynamics, necessary if health systems are to respond to the poor, may be more important than fine-tuning the performance of particular institutional arrangements. Four key developmental perspectives which form an essential part of these broader dynamics are explored below.

#### Social institutional factors

Health systems are complex institutions embedded in rules-based institutional arrangements, but also sustained through social norms and informal practices [39]. Attitudes and understanding of workers in these institutions and those who use them – their framing of problems and solutions – influence performance. Much evidence on the impact of alternative forms of health sector organization comes from advanced market economies, where institutional arrangements and behavioral norms are relatively stable [40]. Health system researchers in these countries have constructed a body of knowledge enabling them to predict the performance of various institutional arrangements.

The situation is different in many low-income countries, where institutions are partially functional and "rules of the game" are changing rapidly [41]. Institutions of the labor market, regulation of professionals and pharmaceuticals, local accountability mechanisms, and the role of the legal system are all in the process of development. Interventions may perform very differently in this context than in mature market economies. Furthermore, expectations and understanding of social system operation vary considerably between stakeholders. These stakeholders, who include citizens, organization managers, and social theorists, are all participating in the creation of new social arrangements; the behavioral norms and expectations of these arrangements are currently being shaped [42]. The language to understand and describe them is also under formation. See Table 1 for key points when considering social institutional factors.

**Table 1:** Social institutional factors – key points

1. Health systems are complex organizations embedded in rules and social norms
2. Human factors within institutions influence processes
3. Rapid change in institutions in low-income countries affect processes
4. Expectations of institutions vary between stakeholders

#### Virtual versus grassroots realities

Marked differences exist between official discourse about health systems and the operational reality in many low-income countries, where a significant proportion of activities take place outside formal rules of national legal frameworks [43]. In the health sector, informal payments and mechanisms to create and preserve reputations and support households facing major medical bills play a very important role. There is little systematic understanding of how these systems work and "policies" take little account of them [39]. There is also little understanding of how different stakeholders envisage their future and how they engage with the politics of health system construction. Influencing agents such as opinion leaders also affect the disconnection between formal rules and reality of decision making. The means through which these influencing agents act vary depending on the type of opinion leader. Thus different types of opinion leaders influence health providers than those that influence household behavior; both are however critical to adoption of knowledge in decision-making and policy implementation.

Many low-income countries receive a proportion of their budgets for health systems from a variety of donor agencies. These agencies play an important role in influencing government systems and in determining the dominant discourse for discussing systems development [44,45]. Reform options are often negotiated between officials in government and donor agencies, while other important stakeholders do not fully participate in these processes. See Table 2 for key points when considering virtual versus grassroots realities.

**Table 2:** Virtual versus grassroots realities – key points

1. Marked difference between theory and practice in health systems operation
2. Informal mechanisms are parallel to formal processes, and are not well understood
3. Analysis of opinion leader functioning is critical
4. Donor agencies influence health systems development, often to the exclusion of other stakeholders but may be non-inclusive

#### Science and society

Much literature exists on the translation of evidence on scientific phenomena into technological changes that influence work and consumption. The history of the knowledge economy points to the close inter-relationship between the growth of basic scientific knowledge, the construction of institutional arrangements to legitimate this knowledge, and a society's capacity to transform technological developments into the creation of a modern economy [46]. The process refers to the existence of a community of educated people, who develop shared understanding of scientific explanations of nature, enabling them to accept technological innovations and fit them into a systematic narrative. This process is underpinned by creation of institutions to support trusted scientific discourse [46].

Nevertheless even in advanced market economies diverse discourses exist surrounding scientific and technical phenomena [47]. In many instances – from controversies over drug and vaccine safety, to the negotiation of appropriate treatment pathways – expert institutions and the scientific perspectives that legitimize them have been critiqued by citizens' and patients organizations [48]. Such critique, and forwarding of alternative perspectives, has been based both on people's own lay knowledge and 'experiential expertise', and on the knowledge of experts in alternative and complementary therapies. In many developing country settings, the plurality of technical knowledge and perspectives is even more pronounced, as a multiplicity of local 'traditional', non-biomedical preventive and therapeutic forms interact with biomedicine in new combinations and hybrids. Thus the argument that shared language and understanding of scientific explanations of nature has become the *norm *needs to be questioned, and qualified by asking who is in this community of shared knowledge, and who is excluded?

See Table 3 for key points when considering science and society.

**Table 3:** Science and society – key points

1. Development of a scientific community is critical to consider
2. Perceptions (and explanations) of science varies in different societies
3. Alternative approaches to health require exploration
4. Inclusion and exclusion from the 'knowledge community' require analysis

#### Construction of social arrangements

Complex social arrangements are emerging in low-income countries in the context of continually negotiated power relationships and inequalities. Esping-Andersen [49] suggests there are different welfare regimes that reflect different understanding of the state's role and different expectations of stakeholders. MacIntosh and Roy [50] argue these different expectations explain the path-dependent nature of social policy. Stability of these regimes and of the attitudes of actors within them has made possible development of increasingly complex institutional arrangements and generation of a body of evidence on how alternative arrangements influence performance. Yet even in advanced market economies, this common understanding may be coming under pressure due to the emergence of new types of institutions, the influence of new medical technologies, new technologies for knowledge management, and the challenge to existing social arrangements from expanded global competition.

The situation is fluid in low-income countries experiencing rapid social change; the roles of government and other regulatory agencies are evolving quickly. This is taking place in the context of rapidly changing patterns of inequality, where stakeholders are renegotiating their position [51]. One of the paradoxes of the health sector and other high trust social enterprises in circumstances of strongly contested power relationships, is that in order for institutions to function well they need high levels of social legitimacy, that mostly imply universal rights and obligations. However, these are superimposed on competing discourses around legitimate and illegitimate inequalities. How can one construct a social consensus to underpin the functioning of complex institutional arrangements in a context of rapid change? How can one enable competition and struggle while constructing high trust institutions? How can one engender the kinds of trust necessary for the functioning of a knowledge economy, while also preserving participation in decision-making that allow different forms of knowledge and ideals concerning nature and social institutions to coexist? These latter requirements may indeed go together, since as recent work suggests, if citizens are to find institutional arrangements and policy processes trustworthy, they have to see in them a capacity to take their concerns seriously [48]. These are critical areas of inquiry for the context within which research and evidence ought to influence policy and practice. See Table 4 for key points when considering construction of social arrangements.

**Table 4:** Construction of social arrangements – key points

1. Social arrangements continuously in flux
2. Inequalities in health, a central consideration, can be seen as legitimate or illegitimate
3. Social legitimacy of policy making is a critical area of enquiry

### Approaches to the research-policy interface

In view of the dynamic context within which health decisions are being taken (described above), an exploration of the research to policy interface becomes more challenging in the developing world. As a consequence, it is conceptually easier to disaggregate the critical aspects of this interface. This section identifies three such areas that provide entry points into the research to policy interface.

#### Recognizing policy as political and complex processes

The traditional model of policy making is a linear process in which rational decisions are taken by those with authority and responsibility for a particular policy area [31]. In this highly-stylized view policy proceeds through a set of stages from understanding the nature of the problem (agenda-setting), to exploring possible problem resolution options, weighing up costs and benefits, making a rational choice about best options (decision-making), and finally implementation, possibly followed by evaluation. 'Evidence' may be called upon at any or all of these stages.

Others argue for a more complex view of policy development and the relationship with knowledge [36]. Bowen and Zwi [52] refer to "pathways to evidence-informed policy and practice", describing a myriad of channels through which evidence influences policy; Buse and colleagues [53] make a similar argument. Keeley and Scoones [35] propose a framework for understanding policy processes concerning the environment, as the interaction of competing interest groups; actors and networks; and policy narratives and discourse. Fairhead and Leach [32] consider relationships between science and policy in developing countries in similar terms. In these frameworks, the process of gathering evidence for policy is envisaged less as the result of a pure and rational quest for what is technically correct – and more about the establishment of 'facts' within particular networks. It is the influence of such networks and their stability in mainstream institutions, nationally and internationally, that is central to their ability to affect policy change.

Shifting the focus of empirical analysis to a process-based view of policy involves recognition that policy-making must be understood as a political process, as much as an analytical or problem solving one. This means that overlapping and competing agendas exist, decisions are not discrete and technical, and that facts and values are intertwined – all variables that characterize the process of decision making.

Policy-making is also complex because it takes place at multiple levels – from international to local. Similarly, implementation of these policies occurs at multiple levels and involves discretion and negotiation at all levels. The perceptions of different officials (both governmental and non-governmental) at various tiers are critical to consider [42]. National officials are often strongly influenced by forward-looking policy debates, projections of future developments, and international experiences. Sub-national officials often tend to respond to local constraints and support local innovations, while being skeptical of the relevance of ideas from the top. There is a clear need to understand how evidence influences decision making at each of these levels and in addition how the levels interact with each other. See Table 5 for key points when considering policy as political and complex processes.

**Table 5:** Policy as political and complex processes – key points

1. Linear versus complex views of policy making processes
2. High level of influence of policy networks
3. Crucial to understand political dimensions of policy making process
4. Multiple tiers of policy making – from international to local

#### Engaging key stakeholders: decision-makers, providers, scientists, and communities

Four key actors are essential to consider when analyzing health sector policy – the government, health providers, scientists, and the community. Bound together through principal-agent relationships and accountabilities, each group has an important role in health research and policy.

A vital element for promoting the use of health research for policy development needs greater attention – the *engagement of decision makers*. There has been a growing realization among both researchers and decision makers that research can improve management decisions and the performance of national health systems [3]. The international development community is now supporting this realization; an example is the Mexico Ministerial Summit on Health Research, which took place in 2004 [54]. However, there is a lack of scientific knowledge on the mechanisms to promote such engagement and their level of success, especially in low-income countries. For example, health policy forums, which have been used in many developed countries, appear not to have been fully evaluated in terms of their potential utility in low-income countries.

Conduct of research or existence of evidence does not guarantee input into the policy development process unless decision makers are appropriately engaged. The process of *translation *of research findings into *pro-policy information *is a critical and yet under-studied process. Informal and formal mechanisms used for such translation and the types of people involved, especially in entities like health policy units, are particularly important to consider. Further, 'demand driven research networks' that respond to national decision maker questions by analyzing and synthesizing available evidence into a user friendly form need to be better explored in a developing world setting. Models for such networks exist in the developed world – for example the Health Evidence Network of WHO Europe [55] – and have the potential for application in low-income countries.

Although global and national efforts have been made to consult with decision makers, *empirical work *in this field is lacking, especially in low-income countries. As a result, there is a need to understand how decision makers view research and what will stimulate them to promote health systems research. Well-developed methodological inquiry to document determinants of decisions, funding flows, and catalysts for policy development are needed. The use of standardized empirical approaches across multiple countries may illuminate understanding of evidence or knowledge valued in various countries, and how particular contexts affect policy development processes.

Scientific experts and decision makers can 'mutually construct' policy, jointly negotiating questions to be answered and types of knowledge to answer them. Scientists can contribute to framing policy issues by defining what evidence can be produced and its policy significance; decision makers can frame scientific enquiry by defining areas of relevance and pertinent areas for investigation. This is sometimes referred to as the co-production of science and policy; thus policy and science are inextricably linked [56]. Should scientists leave policy to policy-makers or should they venture an opinion on policy based on their scientific findings. Arguments have been presented on keeping research findings and policy making separate [57]; the alternative view, that researchers should be unfettered to make policy suggestions, has also been defended [58].

Scientists and decision makers are fundamentally different on various dimensions [13,59]. Recognizing the current schism between these two groups, it is instructive to explore the definition of both policy and science. Policy has been defined as, "a course of action or principle adopted or proposed by a government, party, business, or individual" [60]; science has been defined as "the state of knowing: knowledge as distinguished from ignorance or misunderstanding" [61]. These two definitions do not appear to indicate a clash between science and policy; in fact the two seem complementary. Science need not merely inform policy, but policy-making can itself become a science, and this might have significant implications. Who should these 'policy scientists' be? Is there a need, as some suggest for a new profession of intermediaries between decision makers and research scientists [59]; or can scientists skilled in policy making, and decision makers skilled in public health science take on this role?

The need for continuous exchange of ideas between scientists and decision makers throughout the policy making process has been suggested [62]. Given the importance of communication for effective public health practice [63] this continuous exchange is essential to effective public health policy making. Effective ways of promoting and sustaining such communication need development and testing in low-income countries.

Health providers play a central role in policy implementation, and are often pivotal in the development of new policies. In some cases they share professional interests with the scientific researchers, often sharing similar education and values. In many cases, the stakeholder group most neglected is the community – the beneficiaries of the health system. Health policymaking is incomplete if the focus is solely on government and providers; community participation cannot be overlooked. The examination of how decision makers and researchers in developing countries currently place the role of such approaches, or how communities view the *national *policy making process, is a research agenda. In particular, exploration of how communities affect *local *policy making and implementation, but perhaps more importantly how these local decisions affect national policy may prove particularly enlightening. Further, how these local communities utilize research conducted at local or national levels – and how formal research perspectives articulate with community knowledge and perspectives concerning health issues – warrant empirical consideration. See Table 6 for key points when considering the engagement of key stakeholders.

**Table 6:** Engaging key stakeholders – key points

1. Four key actors: government; providers; scientists; and the community
2. Government decision maker perspectives on evidence translation warrants evaluation
3. Methods of user-friendly packaging of evidence require testing
4. Fundamental differences exist between decision makers and scientists
5. Community perspectives on science and policy making require investigation

#### Enhancing accountability

Accountability refers to one group being responsible for something or to another group, and being able to explain their actions. Policy processes include some perspectives at the expense of others. Much previous research suggests perspectives of the poor and marginalized are often excluded. The role of measuring and monitoring *accountability *in policy proposals and policy implementation warrants careful analysis. Information from low-income countries is particularly scant on such health policy accountability. A promising example comes from the work on benchmarks of fairness for health care reform in several developing countries [64,65]. Such assessment using a broad interpretation of fairness for health sector reform proposals allows a focused evaluation of accountabilities.

The role of *equity analysis *in the research-policy interface needs to be specifically documented; key equity criteria to use in such an analysis have previously been articulated [66]. This may include research on the response of the health system to needs of the poor or specific vulnerable groups. A better understanding of the role of civil society organizations that represent the interests of the poor and the sources of knowledge they use is also needed.

The *human rights *dimensions of health research for policy cannot be ignored. How is this dimension incorporated into health policy formation in low-income countries? Health policies have an undoubted impact on the human rights of particularly vulnerable populations as well as on mainstream populations. Frameworks for the analysis of health policies in terms of human rights ramifications are available [67]. The use of such frameworks may prove an illuminating means of carrying out such an analysis; this may be of critical importance given an often stated focus of health and development policies affecting the poor. See Table 7 for key points when considering the enhancement of accountability.

**Table 7:** Enhancing accountability – key points

1. Perspectives of the poor and marginalized are often excluded from policy making
2. Benchmarks of fairness can be applied to health systems development plans
3. Equity analysis methods can be applied to enhance accountability
4. Frameworks can be utilized to analyze the human rights impacts of health policies

### Frameworks and linkages

**Figure 1:**
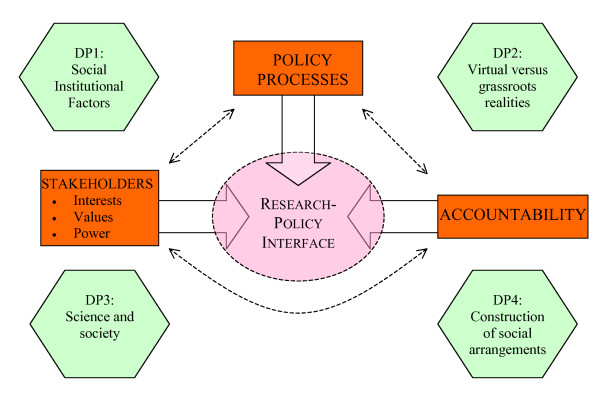
**Proposed conceptual framework: The research-policy interface in low-income countries**. DP = Developmental Perspective.

A discussion of the three entry points within the context of the four developmental perspectives demonstrates the disparate considerations required at the research-policy interface in low-income countries. The complexity of attempts to gain an understanding of this interface becomes apparent. How can these disparate considerations be brought together? A possible approach is presented in the proposed conceptual framework for research-policy interface analysis in low-income countries (Figure 1). Despite the apparent simplicity of the framework each component within the framework is complex, and is described in this paper. Some additional points are made here to add clarity to the framework.

Three entry points to the research-policy interface are presented and these are labeled 'policy processes'; 'stakeholder interests, values, and power'; and 'accountability' (Figure 1). 'Policy processes' includes all the factors discussed in the section on recognizing policy as political and complex processes. 'Stakeholder interests, values, and power' includes the factors discussed in the section on engaging key stakeholders (decision-makers, providers, scientists, and communities). Stakeholders refer to "individuals or groups with a substantive interest in an issue, including those with some role in making a decision or its execution [53]." Stakeholders have "interests", but also have "values" and "power". Values refer to "what we believe in, what we hold dear about the way we live" and these values "influence our behavior as persons, groups, communities, cultures – perhaps as species [68]." 'Accountability' includes all the factors discussed in the section on enhancing accountability and refers to responsibilities governing relationships between stakeholders, and their ability to explain their actions. These three entry points are of course inter-related. They are placed within a context provided by the four developmental perspectives as discussed in the previous section. Context refers to "systematic factors – political, economical, social, or cultural, both national and international – which may have an effect on health policy [53]."

Each of the three entry points affects the research-policy interface, represented by the box at the center of the framework (Figure 1). The three entry points as well as the interface itself are affected by the four developmental perspectives. As the entry points converge on the 'interface box' and merge with one another it becomes evident that many of the issues can be placed in more than just one of the conceptual groupings. It remains illuminating however, to carry out such conceptual grouping in order to increase clarity in examining the distinct types of influences on the research-policy interface. This may aid in both retrospective and prospective analysis of the decision making process.

### Responding to gaps in knowledge on the research-policy interface

Understanding critical issues in the relationship between research and policy, together with an appreciation of the context is a prerequisite to further investigation of the interface. An exploration of the interface based on the proposed conceptual framework, can use a wide variety of approaches; these are described below. This section on theory forms the basis of empirical work to be conducted by FHS country teams and aims to highlight important methodological issues.

#### Potential study designs

A number of different types of designs can be used in exploring the research-policy interface. These include: policy focused approaches; research focused approaches; and cross sectional approaches. *Policy focused *approaches involve selection of a specific national (or local) policy and retrospectively tracing the determinants of policy development or its implementation. In particular, the forms of knowledge, actors, networks, and interests involved in policy development can be examined using this approach. When considering policy development this approach examines matters in terms of people, evidence, and processes; such a design attempts to develop an *explanatory framework *for the existence of a specific health policy in the hope of learning if that framework can be generalized to other instances. When policy implementation is the focus, exploration hones in on the process used to implement a policy over a few years and documents the role of research in its success (or failure) [15].

*Research focused *approaches are a much more challenging and time consuming design. The approach prospectively follows a body of evidence and seeks to track the impact of the evidence on the development or refinement of policy at national or local level. The expectation of this design is a body of relevant and well-developed research should impact on policy at national level, and studying that process will allow key determinants of such a flow to be monitored and documented [69].

Finally, *cross sectional *approaches are the simplest design and documents existing processes in the research-policy interface. This approach helps to identify research priority selection methods, mechanisms to translate research to policy, and effects of policy demands on research agendas; either policy or research is a starting point for inquiry [70].

#### Potential methodologies

A number of specific methods can be used to explore the research to policy interface. They include: surveys; in-depth or semi-structured interviews; focus group discussions; document reviews; news/media analysis; and a variety of participatory methods using visual approaches such as institutional mapping. *Surveys *can be used to seek specific information regarding the research-policy interface based on a structured interview format. These can be applied to individuals (most common) or institutions, and usually involve closed-ended questions and can cover a breadth of issues [15]. *In-depth or semi-structured interviews *offer a more open ended version to seek information from individuals usually focusing on a few areas. When they are used in earlier phases to guide further explorations or when they are targeted to specific individuals, they are referred to as '*key informant*' interviews [71]. *Focus group discussions *offer a discussion format to identify common perspectives on key issues and are very useful to either provide a broad coverage of issues or test out ideas (such as potential interventions). *Document reviews *provide an opportunity for analysis of existing literature especially government reports, policy briefs, statements and unpublished documents [70]. *News and media analysis *has been used to understand how the media reports both research and policy issues. This has also been used to explore the 'public understanding of science'. Finally, a range of methods drawing on the broader field of participatory research and appraisal [72] involves engaging policymakers and researchers in reflection on the nature of policy processes and the influences on them. Tools such as the visual mapping of institutions and actor-networks, timelines and event histories have also been usefully incorporated into such processes [73].

Research involving decision makers warrants specific consideration. First, since decision makers are very busy and unlikely to give extended time, methodological options are often limited. Second, the key to such exploration is to understand decision maker attitudes and perspectives, and therefore opportunities for them to provide such views needs to be prioritized; interestingly, decision makers often find it refreshing and illuminating to reflect on processes they may have taken for granted. Third, in view of the above circumstances, a mix-methods approach is often used involving both qualitative and quantitative methods. Fourth, *levels of analysis *need to be defined (for example a focus on national, state, or district level decision makers); this should not however preclude analysis at multiple levels, tracking policy processes and actor-networks across these levels. Lastly, a *decision maker *needs to be carefully defined (for example as an individual who has *decision making authority *or authority to allocate funds). Illustrative guides to the types of decision makers that need to be engaged at national and local levels are listed in Table 8.

**Table 8:** Types of decision makers at national and local level (illustrative only)

**NATIONAL**	**LOCAL**
**GOVERNMENT** **Ministry of Health: **Minister; Secretary; Director-General; Assistant Director General for Research **Heads of National Institutes: **Health; Public Health; Statistical; Demographic; Medical Research **National Assembly/Parliament: **Prime Minister or President; Head of Standing Committee on Health **Ministry of Finance and/or Planning: **Minister; Secretary; Director-General; Head of Health Sector **Ministry of Science and Technology: **Minister; Secretary; Director-General; Head of Health Affairs **Ministry of Education: **Minister; Secretary; Director-General; Head of Health and Medical Section	**GOVERNMENT** **Health Administration: **Head of Health Authority; Hospital Chief Executive; Head of District Health Center **Public Administration: **Head of Local Government Authority; Head of Finance Department; Head of Education Department
**PRIVATE FOR PROFIT** Head of hospital associations; Head of pharmaceuticals; Health insurance companies	**PRIVATE FOR PROFIT** Head of companies with health schemes; Private health care providers
**CIVIL SOCIETY** NGO/PVO Head/Chief of Party	**CIVIL SOCIETY** Head of local NGO/PVO; Traditional and religious leaders; Union leaders; Community leaders
**HEALTH CARE PROVIDERS** Head of medical, nursing, and other professional associations	**HEALTH CARE PROVIDERS** Doctors; Nurses; Community Health Workers; Pharmacists; Traditional Healers

#### Application of theory to practice

Clear gaps in current literature are cross-sectional, multi-country, collaborative studies ensuring comparability of methods; this could potentially be a great step forward in this field of inquiry. Six country teams within the FHS research consortium are utilizing approaches summarized in this paper to enhance knowledge on research-policy interface strengthening. Research activities in one partner country – Bangladesh – are described to elucidate how the presented theory is being used in practice.

The FHS country team in Bangladesh has chosen to explore the informal health care system in a rural area of Bangladesh. This is an innovative area of work looking outside the confines of formal health systems and is thus in keeping with the consortium focus on innovations. The proposed framework has been utilized to highlight key areas requiring exploration. Each of the four developmental perspectives has contributed to the proposed research. First, social institutional factors highlight the importance of exploring rapidly changing complex rural health organizations (by conducting institutional analysis); the interface between formal and informal institutions; and the expectations that stakeholders have of these institutions. Second, considering differences in virtual versus grassroots realities highlights the importance of information gathering from rural users of the informal health system; increasing understanding of opinion leaders in rural settings; and exploring donor agency perspectives on incorporating the informal health sector in health systems development. Third, the interaction of science and society is being explored in depth to determine population perceptions of the science underpinning formal and informal health care systems and the determinants of alternative approaches to health. Lastly, the construction of social arrangements is being explored in a rural setting – the health seeking behavior of the poor is focused upon.

Considering the three entry points within the framework allows focus on pertinent areas of investigation. Policy processes are being examined with a special focus on the local rural political landscape as well as influential decision-maker networks. Stakeholder interests, values, and power are being explored through formal stakeholder analyses – this analysis covers a wide spectrum of stakeholders including the government (at multiple levels), providers, researchers, and the community. Issues related to accountability are being explored by focusing on the perspectives of poor and marginalized beneficiaries.

Study designs and methods described in this section are being utilized by the FHS Bangladesh country team – while explorations are at an early stage, the framework has provided a sound grounding for activities. As Bangladesh and the other five FHS country teams begin to report their findings it is envisioned that the conceptual framework will be tested and further refined.

## Conclusion

The rapidly evolving nature of the research to policy interface within low-income country settings necessitates substantial use of novel approaches to respond to this evolution. These approaches include: utilization of a conceptual framework of the interface that highlights the importance of particular entry points and appreciates essential developmental perspectives; new methodological techniques in analyzing the interface; choice of research topics with significant scope for innovation; and creative partnership formation to carry out the analysis. Many sources of innovations currently influence health systems. Some arise from importation of organizational arrangements from other societies; policy leaders largely introduce these. Others emerge as local adaptations to local challenges. A third source is new technologies and new organizational forms. These new technologies may play a more dynamic role in rapidly changing societies than in ones with well-established welfare regimes.

Analyses of current flow of innovations into the health system are instructive to health systems development. Many ideas for reform are transmitted through international agencies. There is a need to explore how these agencies perceive their role in knowledge transmission. There is also need to carefully explore emerging local innovations and how information about them is diffused; documenting different understandings of the purpose of these innovations and their performance is essential. Instances where importation of ideas has led to successful national innovations need examination. In particular, examples of the market creating innovative organizational arrangements require careful analysis.

Research conducted by Future Health Systems represent a great opportunity to develop an empirical basis for understanding how decision makers perceive research, and what value they ascribe to it in terms of their own decision making in the health sector. Such projects can also illuminate attractive research characteristics from a decision maker perspective and thus suggest types of research these decision makers are likely to commission. Empirical work can allow a powerful qualitative comparison of similarities and differences in the perspective of decision makers across countries and issues. It may also help to define how global programs can promote health research to decision makers and what generic characteristics of research make it useful for decision-making. Such empirical studies will be innovative in low-income countries in focusing on decision maker's perspective towards health research utilizing standard methods and a cross-sectional, multi-country approach. The developmental context incorporated into the entire endeavor can significantly enrich the findings. A vision of health system development cognizant of the myriad factors at the research-policy interface can establish the basis for future health systems.

## Competing interests

The author(s) declare that they have no competing interests.

## Authors' contributions

AH conceived the idea of the paper in collaboration with GB. ML assisted this conception phase of the paper. SBS added sections to the paper and refined the conceptual framework. DHP reviewed and edited the paper. All authors were involved with reviewing the paper. The last author, FHS itself, recognizes the contribution of all the professionals within the research consortium. All authors read and approved the final manuscript.

## Pre-publication history

The pre-publication history for this paper can be accessed here:



## References

[B1] Global Forum for Health Research (2000). The 10/90 report on health research.

[B2] Nchinda TC (2002). Research capacity strengthening in the South. Soc Sci Med.

[B3] World Health Organization (2004). World report on knowledge for better health: strengthening health systems.

[B4] Global Forum for Health Research (1999). The 10/90 report on health research.

[B5] Commission on Health Research for Development (1990). Health research: essential link to equity in development.

[B6] Global Forum for Health Research (2004). Monitoring Financial Flows for Health Research.

[B7] Pang T, Sadana R, Hanney S, Bhutta ZA, Hyder AA, Simon J (2003). Knowledge for better health: a conceptual framework and foundation for health research systems. Bull World Health Organ.

[B8] Haines A, Kuruvilla S, Borchert M (2004). Bridging the implementation gap between knowledge and action for health. Bull World Health Organ.

[B9] DeRoeck D (2004). The importance of engaging policy-makers at the outset to guide research on and introduction of vaccines: the use of policy-maker surveys. J Health Popul Nutr.

[B10] Hall W (2004). The contribution of research to Australian policy responses to heroin dependence 1990–2001: a personal retrospection. Addiction.

[B11] Kindig D, Day P, Fox DM, Gibson M, Knickman J, Lomas J, Stoddart G (2003). What new knowledge would help policymakers better balance investments for optimal health outcomes?. Health Serv Res.

[B12] Lavis JN, Posada FB, Haines A, Osei E (2004). Use of research to inform public policymaking. Lancet.

[B13] Brownson RC, Royer C, Ewing R, McBride TD (2006). Researchers and policymakers travelers in parallel universes. Am J Prev Med.

[B14] Dandona L (2002). Conceptualizing health policy. Natl Med J India.

[B15] Tomson G, Paphassarang C, Jonsson K, Houamboun K, Akkhavong K, Wahlstrom R (2005). Decision-makers and the usefulness of research evidence in policy implementation – a case study from Lao PDR. Soc Sci Med.

[B16] Stockwell A, Whiteford H, Townsend C, Stewart D (2005). Mental health policy development: case study of Cambodia. Australas Psychiatry.

[B17] Harpham T, Tuan T (2006). From research evidence to policy: Mental health care in Viet Nam. Bull World Health Organ.

[B18] Thamlikitkul V (2006). Bridging the gap between knowledge and action for health: Case studies. Bull World Health Organ.

[B19] Shiffman J, Wu Y (2003). Norms in tension: democracy and efficiency in Bangladeshi health and population sector reform. Soc Sci Med.

[B20] Campbell BB, Reerink IH, Jenniskens F, Pathak LR (2003). A framework for developing reproductive health policies and programmes in Nepal. Reprod Health Matters.

[B21] Hornby P, Perera HS (2002). A development framework for promoting evidence-based policy action: drawing on experiences in Sri Lanka. Int J Health Plann Manage.

[B22] Kapiriri L, Norheim OF, Heggenhougen K (2003). Using burden of disease information for health planning in developing countries: the experience from Uganda. Soc Sci Med.

[B23] Ashford LS, Smith RR, De Souza RM, Fikree FF, Yinger NV (2006). Creating windows of opportunity for policy change: Incorporating evidence into decentralized planning in Kenya. Bull World Health Organ.

[B24] Richards FO, Eigege A, Miri ES, Jinadu MY, Hopkins DR (2006). Integration of mass drug administration programmes in Nigeria: The challenge of schistosomiasis. Bull World Health Organ.

[B25] Albert MA, Fretheim A, Maiga D (2007). Factors influencing the utilization of research findings by health policy-makers in a developing country: the selection of Mali's essential medicines. Health Res Policy Syst.

[B26] Trostle J, Bronfman M, Langer A (1999). How do researchers influence decision-makers? Case studies of Mexican policies. Health Policy Plan.

[B27] World Health Organization. http://www.who.int/en/.

[B28] Alliance for Health Policy and Systems Research. http://www.alliance-hpsr.org/jahia/Jahia/.

[B29] Council on Health Research for Development. http://www.cohred.org/main/.

[B30] COHRED Working Group on Research to Action and Policy (2000). Lessons in Research to Action and Policy: Case studies from seven countries.

[B31] Alliance for Health Policy and Systems Research (2004). Strengthening health systems: the role and promise of policy and systems research.

[B32] Fairhead J, Leach M (2003). Science, society and power: environmental knowledge and policy in West Africa and the Caribbean.

[B33] Crewe E, Young J (2002). Bridging Research and Policy: Context, Evidence, and Links.

[B34] Oliver TR (2006). The politics of public health policy. Annu Rev Public Health.

[B35] Keeley J (2003). Understanding environmental policy processes: cases from Africa.

[B36] Walt G (1994). Health policy: an introduction to process and power.

[B37] Peters P, Bloom G, Rahman MH, Bhuiya A, Kanjilal B, Oladepo O, Pariyo G, Zhenzhong Z, Sundaram S, Global Forum for Health Research (2006). Research for future health systems. The Global Forum Update on Research for Health.

[B38] Future Health Systems: Innovations for Equity. http://www.futurehealthsystems.org/index.htm.

[B39] Bloom G, Lloyd R, Standing H (2006). Rethinking future health systems.

[B40] Bloom G (2004). Private Provision in its institutional context: Lessons from Health.

[B41] McPake B, Mills A (2000). What can we learn from international comparisons of health systems and health system reform?. Bull World Health Organ.

[B42] Bloom G, Gauld R (2005). Health policy during China's transition to a market economy. Comparative health policy in the Asia-Pacific.

[B43] Duffield MR (2001). Global governance and the new wars: the merging of development and security.

[B44] Walt G, Merson MH, Black RE, Mills A (2001). Global Cooperation in International Public Health. International public health: diseases, programs, systems, and policies.

[B45] Walt G (2005). What sort of international cooperation in health 2055?. J R Soc Med.

[B46] Mokyr J (2002). The gifts of Athena: historical origins of the knowledge economy.

[B47] Gaskell G, Einsiedel E, Hallman W, Priest SH, Jackson J, Olsthoorn J (2005). Communication. Social values and the governance of science. Science.

[B48] Leach M, Scoones I, Wynne B (2005). Science and citizens: globalization and the challenge of engagement.

[B49] Esping-Andersen G (1990). The three worlds of welfare capitalism.

[B50] Mackintosh M, Roy R (1999). Economic decentralization and public management reform.

[B51] Bloom G (2001). Equity in health in unequal societies: meeting health needs in contexts of social change. Health Policy.

[B52] Bowen S, Zwi AB (2005). Pathways to "evidence-informed" policy and practice: a framework for action. PLoS Med.

[B53] Buse K (2005). Making health policy.

[B54] Bell S (2005). From practice research to public policy – the Ministerial Summit on Health Research. Ann Pharmacother.

[B55] Dumitrescu A, Granados A, Wallace J, Watson S (2006). Demand-driven evidence network in Europe. Bull World Health Organ.

[B56] Briss PA, Gostin LO, Gottfried RN (2005). Science and public health policy makers. J Law Med Ethics.

[B57] Rothman KJ, Poole C (1985). Science and policy making. Am J Public Health.

[B58] Teret S (2001). Policy and science: should epidemiologists comment on the policy implications of their research?. Epidemiology.

[B59] Choi BC, Pang T, Lin V, Puska P, Sherman G, Goddard M, Ackland MJ, Sainsbury P, Stachenko S, Morrison H, Clottey C (2005). Can scientists and policy makers work together?. J Epidemiol Community Health.

[B60] Oxford University Press (1998). The new Oxford dictionary of English.

[B61] Merriam-Webster Online Dictionary. http://www.m-w.com/.

[B62] Samet JM, Lee NL (2001). Bridging the gap: perspectives on translating epidemiologic evidence into policy. Am J Epidemiol.

[B63] Bernhardt JM (2004). Communication at the core of effective public health. Am J Public Health.

[B64] Daniels N, Bryant J, Castano RA, Dantes OG, Khan KS, Pannarunothai S (2000). Benchmarks of fairness for health care reform: a policy tool for developing countries. Bull World Health Organ.

[B65] Daniels N, Flores W, Pannarunothai S, Ndumbe PN, Bryant JH, Ngulube TJ, Wang Y (2005). An evidence-based approach to benchmarking the fairness of health-sector reform in developing countries. Bull World Health Organ.

[B66] James C, Carrin G, Savedoff W, Hanvoravongchai P (2005). Clarifying efficiency-equity tradeoffs through explicit criteria, with a focus on developing countries. Health Care Anal.

[B67] Gostin L, Mann JM (1994). Towards the development of a human rights impact assessment for the formulation and evaluation of public health policies. Health Hum Rights.

[B68] Last JM, International Epidemiological Association (2001). A dictionary of epidemiology.

[B69] Kroeger A, Hernandez JM (2003). Health services analysis as a tool for evidence-based policy decisions: the case of the Ministry of Health and Social Security in Mexico. Trop Med Int Health.

[B70] Kivumbi GW, Kintu F (2002). Exemptions and waivers from cost sharing: ineffective safety nets in decentralized districts in Uganda. Health Policy Plan.

[B71] Dangour AD, Moreno X, Albala C, Rivera-Marquez A, Lera L, Villalobos A, Morris SS, Uauy R (2005). Chile's national nutritional supplementation program for older people: lessons learned. Food Nutr Bull.

[B72] Chambers R (1997). The origins and practice of participatory rural appraisal. World Development.

[B73] Wolmer W (2006). Understanding Policy processes: A review of IDS research on the environment.

